# Prescribed Drugs and Interpersonal Violence: A Case–Non-Case Study in the Spanish Pharmacovigilance Database

**DOI:** 10.3390/ph18121845

**Published:** 2025-12-03

**Authors:** Ana Avedillo-Salas, Ana Fanlo-Villacampa, Francisco Javier Lanuza-Giménez, Jorge Vicente-Romero

**Affiliations:** Department of Pharmacology, Physiology and Legal and Forensic Medicine, Faculty of Medicine, University of Zaragoza, 50009 Zaragoza, Spain

**Keywords:** adverse drug reactions, interpersonal violence, Pharmacovigilance, FEDRA, MedDRA

## Abstract

**Background/Objectives:** Interpersonal violence is an increasing public health concern, and its prediction and prevention remain global challenges. This study aimed to identify prescribed medications associated with interpersonal violence in Spain. **Methods**: A descriptive, longitudinal and retrospective study and case-non case study of spontaneous reports of adverse drug reactions corresponding to interpersonal violence recorded in the Spanish Pharmacovigilance Database (FEDRA^®^) from 1984 to 31 March 2021. **Results**: 533 cases were reported in the study period. The mean age was 46.70 years with ages ranging from 1 to 99 years. There were no sex differences except in child and adolescent age group where most reports were from male. Main therapeutic groups involved were nervous system (62.3%), anti-infectives for systemic use (10%) and respiratory system (8.6%). Mostly drugs reported were montelukast, levetiracetam, bupropion, donepezil, perampanel, quetiapine, fluoxetine, and lorazepam. A statistically significant association/disproportion in the notification has been found in the reporting of interpersonal violence and different drugs according to the literature, notably atomoxetine, perampanel, memantine, donepezil, montelukast and methylphenidate. **Conclusions**: The results highlight that interpersonal violence, while rare, could occur as a clinically relevant adverse reaction to a small subset of medications. They underscore the importance of careful prescribing, especially in vulnerable populations and in individuals with a history of psychiatric disorders.

## 1. Introduction

### 1.1. Background

**Figure 1:**
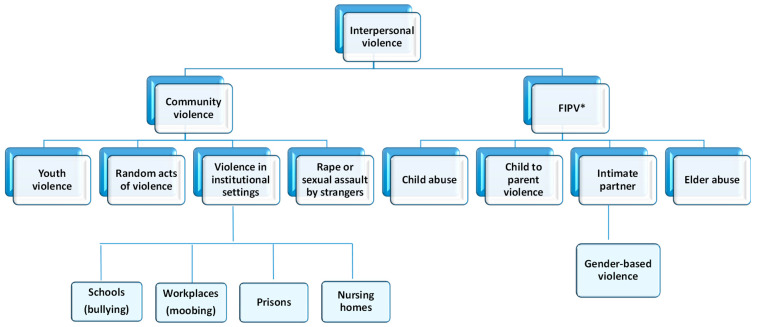
Interpersonal violence classification. * FIPV: Family and intimate partner violence.

The World Health Organization (WHO) defines interpersonal violence as “violence perpetrated by an individual or small group of individuals against another person or persons” [1,2]. This type of violence is a multifactorial phenomenon, which can be classified into different categories depending on the context and the actors involved. The categories encompass community violence and family and intimate partner violence (Figure 1).

The term ‘family and intimate partner violence’ refers to any act or omission by one or more family members against another member in a position of greater vulnerability, resulting in physical, psychological, sexual, economic, or social harm [2,3,4]. This abuse of power primarily impacts vulnerable groups, including children, women, older adults, and individuals with disabilities. It encompasses a number of forms of abuse, including elder abuse [5,6] and child abuse, which may involve physical violence (corporal punishment), sexual violence (incest), or psychological violence [7,8]. It also includes child-to-parent violence, conceptualised as physical, verbal, or non-verbal aggression by children toward their parents or guardians [9,10,11], and intimate partner violence, which involves any violent behaviour by a current or former partner against the other. Within this category, gender-based violence is of particular significance. It is understood as a manifestation of discrimination and inequality perpetrated by men against women within emotional or intimate relationships [12,13,14].

Conversely, community violence transpires among individuals who are not related or in a close relationship, predominantly occurring outside the home, although other forms of social connections may be present. This category encompasses youth violence, defined as acts of violence perpetrated by individuals aged 10 to 29 years [7,15], as well as random acts such as homicide, rape or sexual assault, and violence in institutional settings including schools, workplaces, prisons, and nursing homes. In the contemporary social context, forms of bullying and cyberbullying, mobbing, violence against women and children, and abuse of older adults by individuals outside the family are of particular concern due to their prevalence and societal impact.

In response to the mounting global prevalence of interpersonal violence, the World Health Organization (WHO) adopted a Global Plan of Action in 2016, with the objective of enhancing the capacity of health systems to address violence, with particular emphasis on the prevention and response to violence against children, women, and girls (WHA67.15) [7].

Interpersonal violence is defined as the occurrence of homicidal thoughts, communications, and actions, with consideration given to the outcomes, severity, and the perpetrators’ awareness of their behaviour. Interpersonal violence can manifest in a variety of forms, including physical, psychological, financial abuse, and deprivation or neglect [1,2].

### 1.2. Interpersonal Violence: A Public Health Problem

Interpersonal violence is a grave global public health concern, and its prediction and prevention continue to represent significant challenges for the international community [1,2]. On a global scale, the annual mortality rate attributable to interpersonal violence is estimated at 8.8 deaths per 100,000 inhabitants, while the rate of non-fatal injuries resulting from such violence is substantially higher [2]. Recent studies have shown that the global homicide rate has remained relatively stable over the past two decades, with an annual range of between 400,000 and 450,000 deaths [16].

In the period between 2019 and 2021, approximately 22% of homicides worldwide were associated with criminal groups, whereas 31% were linked to partners or family members [16]. In Europe, the majority of homicides (69%) were committed by family members or intimate partners, while 20% involved other individuals, and only 11% were related to organised crime, other criminal activities, or socio-political causes [16]. In Spain, the homicide rate is recorded at 0.61 per 100,000 inhabitants, which is notably below the European average [17].

Despite Spain’s relatively low homicide rate, the country presents a higher proportion of homicides perpetrated by intimate partners or family members compared with other nations [16,18]. On a global scale, women are disproportionately affected by homicide within the domestic environment, accounting for 55% of victims, compared with 12% of men [19]. A similar pattern is observed across Europe, where 445 women lost their lives to intimate partner or family-related violence in 2022 [19]. It is therefore recognised that the domestic environment represents the primary setting in which women are most at risk of homicide [16]. In Spain, for instance, 3997 homicides were recorded between 2009 and 2020, of which 750 were committed by a partner or ex-partner; notably, 670 of these victims were women [20]. Moreover, in 2022, 176,380 women were officially registered as victims of gender-based violence, with 182,073 complaints filed [21]. Recent studies indicate that an average of 52 women per year are killed by their current or former partners [22,23]. Nevertheless, there remains a paucity of data concerning violent acts perpetrated against male partners, children, and elderly individuals within the family context.

The issue of violence in educational settings is a significant public health and social concern. Globally, it is estimated that 246 million children and adolescents experience violence or harassment at school, whether from classmates, teachers, or other members of the school community [24]. A survey administered by UNICEF in 18 countries revealed that face-to-face bullying is more prevalent in primary education and decreases by approximately 50% between the ages of 14 and 18. Conversely, cyberbullying increases during adolescence [25]. In Spain, 23.3% of students report having been subjected to bullying of some kind during their school years. The consequences for victims are profound, with over half exhibiting symptoms of post-traumatic stress (53.7%), depression (54.8%), and low self-esteem (53%) [26]. Recent studies indicate that 9.53% of primary school students have been subjected to bullying, with a further 9.2% encountering cyberbullying. Additionally, 4.58% of students admitted to bullying their classmates, while 4.62% reported engaging in cyberbullying [27].

Elder abuse represents a significant manifestation of interpersonal violence. The WHO estimates that one in six people over the age of 60 experiences some form of abuse, with a global prevalence of 16% in community settings—a figure expected to rise to 320 million victims by 2050 if current trends persist [6]. A meta-analysis encompassing 28 countries revealed that 15.7% of older adults have been subjected to abuse, including psychological (11.6%), financial (6.8%), neglect (4.2%), physical (2.6%), and sexual (0.9%) violence [28].

Collectively, these findings emphasise that interpersonal violence has a pervasive impact on both the Spanish and global populations, manifesting in diverse forms of abuse across the lifespan. Whilst mortality is undoubtedly the most severe, albeit infrequent, consequence, survivors frequently suffer from chronic physical, functional and psychological sequelae. These sequelae contribute to a substantial societal burden of morbidity and mortality.

It is evident that the World Health Organization (WHO) has identified the reduction in interpersonal violence as a global health priority, as reflected in several strategic frameworks, including the Mental Health Action Plan 2013–2022 (recently extended to 2030) and the INSPIRE strategy, which provides evidence-based guidance for prevention and response efforts worldwide [29,30]. The World Health Organisation (WHO) has established an objective to reduce the global prevalence of recent intimate partner violence from 20% to 15%, with an initial target date of 2023 and a subsequent extension to 2025 [31,32].

### 1.3. Risk Factors

Interpersonal violence is a multifaceted phenomenon, influenced by a wide range of social, psychological, biological, economic, and cultural factors [2,5,7,33]. The emergence of violent behaviour may be attributed to a multitude of risk factors, including individual characteristics such as a documented history of aggression or abuse, mental illness, financial or occupational challenges, and substance use. Interpersonal factors also play a pivotal role, encompassing experiences such as child abuse, elder abuse, gender-based violence, and exposure to violent behaviour by others. At the community level, elements such as bullying, cyberbullying, limited access to healthcare, and sexual violence further exacerbate vulnerability. Furthermore, sociocultural norms that perpetuate violence, reinforce male dominance, and stigmatise mental illness have been demonstrated to inhibit help-seeking behaviours among those affected [1,2,24,29,34].

In the context of these determinants, a particular factor has received comparatively little attention: namely, the utilisation of medications [35,36,37]. Despite the existence of specific pharmaceuticals that explicitly delineate aggressive or violent behaviour as a potential adverse drug reaction in their Summary of Product Characteristics (SmPC), scientific investigation in this domain remains underdeveloped. The association between interpersonal violence and substance use is well established for alcohol and illicit drugs such as cannabis and synthetic “designer” drugs, with robust evidence supporting these links [38,39,40,41,42,43]. In contrast, when considering prescription medications, this association is less evident and has been primarily reported for psychotropic agents. The pharmacological mechanisms most frequently implicated involve decreased serotonin activity, reduced gamma-aminobutyric acid (GABA) transmission, and increased dopaminergic activity. These have been associated with the pathophysiology of aggression and violent behaviour [44,45].

### 1.4. Aim

In order to investigate the little-known role of drugs in interpersonal violence, the objective of this study was to analyse interpersonal violence as an adverse drug reaction, at the national level, based on spontaneous reports of suspected adverse drug reactions from the Spanish Human Pharmacovigilance System Database (FEDRA^®^) from 1984 to 31 March 2021.

## 2. Results

In the study period, the Spanish Human Pharmacovigilance System received 353,165 spontaneous ADRs, of which 533 are spontaneous ADRs of interpersonal violence (0.15%).

### 2.1. General Data on Reports

**Figure 2:**
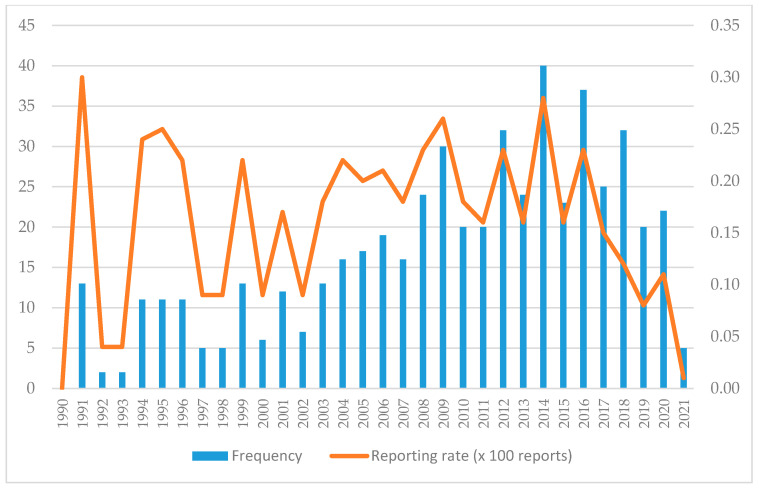
Temporal evolution of reports of interpersonal violence.

Most of the 533 reports were received since 2008 (N = 354; 66.4%), with 2014 (N = 40; 7.5%) and 2016 (N = 37; 6.9%) standing out in particular. However, the years with the highest reporting rates were 1991 and 2014. In addition, there has been a gradual decline in both the number of reports and the reporting rate since 2014 (Figure 2).

The percentage of out-of-hospital reports (n = 332; 60.8%) was higher than in-hospital (n = 145; 26.6%). The professionals who reported the most were physicians (n = 361; 66.1%) and pharmacists (n = 116; 21.2%).

**Figure 3:**
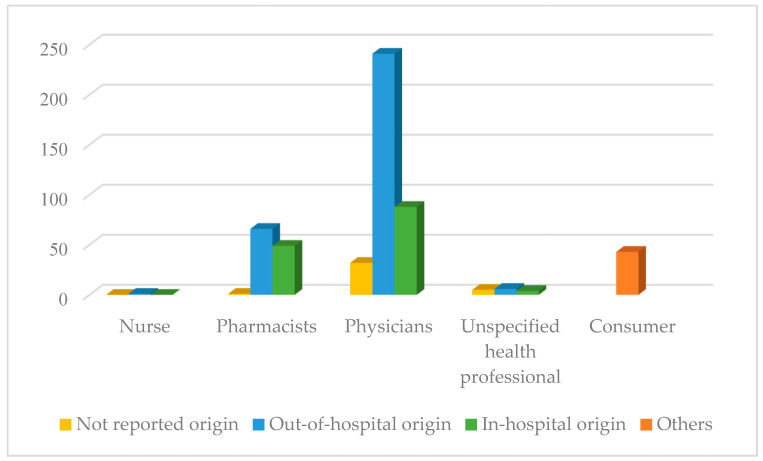
Number of reports according to primary sources.

Analysing the origin of the notifier, in-hospital pharmacists reported 42.20% (n = 49) of the cases compared to 56.90% (n = 66) of out-of-hospital pharmacists and 0.90% (n = 1) was no reported. For their part, out-of-hospital physicians reported 66.80% (n = 241), in-hospital physicians reported 24.40% (n= 88), and not reported 8.9% (n = 32), as shown in Figure 3.

### 2.2. Patient Data (Study Population)

**Figure 4:**
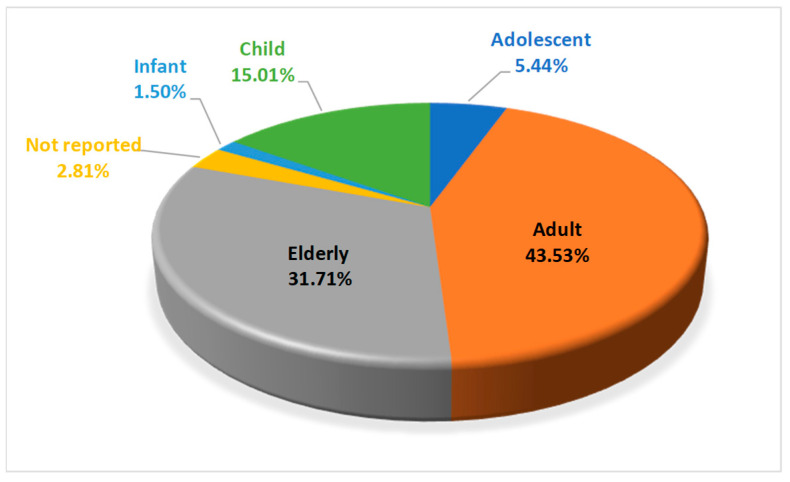
Percentage of interpersonal violence reports by age group.

The mean age was 46.70 years with a standard deviation of 28.07 years with ages ranging from 0.3 to 99 years. The highest number of reports was in the adult group (n = 232; 43.5%) and elderly group (n = 169; 31.7%) (Figure 4).

**Figure 5:**
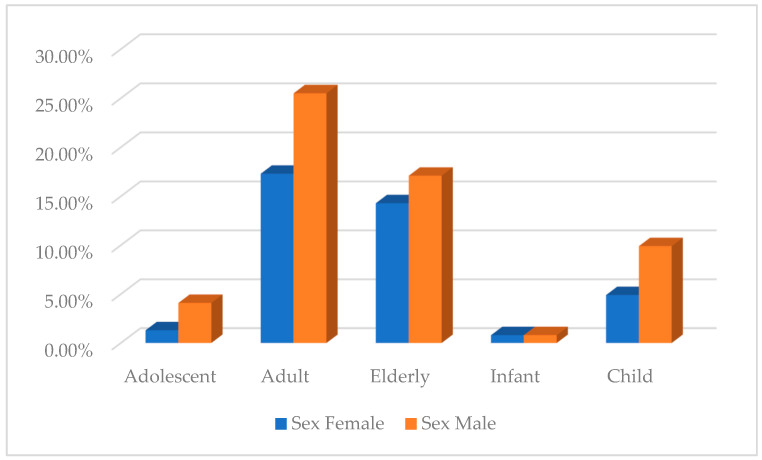
Percentage of interpersonal violence reports by age group and sex.

The distribution of reports according to sex showed that 208 (39%) were female and 315 (59.1%) were male. However, 10 of them did not include sex (1.9%). Figure 5 shows the percentage considering age group and sex, where differences were observed in the child age group, with 66.3% (n = 53) belonging to the male sex compared to 32.5% (n = 26) of the female sex. Likewise, in the adolescent group, 75.9% of cases are male sex.

### 2.3. Interpersonal Violence Adverse Drug Reactions

Out of 533 cases, 300 cases were serious (56.29%) and 233 were no serious. These contained 554 reactions of interpersonal violence of which 3 (0.5%) were fatal, 35 (6.6%) were life-threatening, 65 (12.2%) resulted in hospital admission, 5 (0.9%) prolonged hospital stay, 2 (0.4%) resulted in patient disability and 207 (38.8%) resulted in a medically significant illness.

The most frequently reported suspected ADRs were for PT aggression (480; 86.6%) and behavioural disturbance (35; 6.3%) (Table 1), HLT behavioural or socialisation disturbances (499; 90.1%) and SOC psychiatric disorders (554; 100%).

In addition, 63.7% of cases (n = 353) recovered and 8.5% (n = 47) not recovered (Table 2).

### 2.4. Drugs Involved

In the 533 notifications, 671 suspected drugs were recorded; 49 of them were interactions. The most frequent therapeutic groups to which the suspected drugs belong are the pharmacological groups of the nervous system (n = 419; 62.3%), anti-infectives for systemic use (n = 67; 10%), respiratory system (n = 57; 8.6%), antineoplastic and immunomodulatory agents (n = 26; 3.9%) and alimentary tract and metabolism (n = 25; 3.2%) (Table 3).

Within the nervous system, the therapeutic subgroups of psychoanaleptics (n = 126; 18.8%), psycholeptics (n = 105; 15.3%) and antiepileptics (n = 84; 12.8%) were the most reported (Table 4). The active substances levetiracetam (n = 23; 3.4%), bupropion (n = 18; 2.7%), donepezil (n = 14; 2.1%), fluoxetine (n = 13; 1.9%), quetiapine (n = 13; 1.9%) and perampanel (n = 13; 1.9%) predominate.

Also, among anti-infectives for systemic use are antivirals (n = 30; 4.5%), especially ribavirin (n = 6; 1%) and antibacterials (n = 34; 5.2%), mainly macrolides (n = 15; 2.2%) and fluoroquinolones (n = 8; 1.2%).

Within the respiratory system, antihistamines for systemic use (n = 8; 1.2%) and agents against obstructive airway diseases (n = 41; 6.1%), notably montelukast (n = 35; 5.2%), were the most frequently reported (Table 5).

Likewise, the most reported antineoplastic and immunomodulatory agents were the immunostimulants interferon alfa-2B (n = 4; 0.6%), peginterferon alfa-2B (n = 3; 0.5%) and the immunosuppressant adalimumab (n = 3; 0.5%). Table 5 provides a list of the drugs most frequently implicated in interpersonal violence adverse drug reactions. The complete list of interpersonal violence reports for each of the 251 drugs can be found in Supplementary Materials.

At the top of the list, with 35 reports of adverse drugs reactions was montelukast implicated in 32 cases of aggressions, 2 cases of behavioural disturbance, one case of hostility, and one case of antisocial behaviour. Of these, 23 occurred in children, six in adolescents, and one in an infant. In terms of sex, 29 were in males and six in females. It was followed by levetiracetam implicated in 23 cases: 20 aggressions, one homicidal ideation, two behavioural disturbances and one case of hostility, with two occurring in adolescents and one in a child.

This list includes the selective catecholamine (norepinephrine and dopamine) reuptake inhibitor bupropion, which was reported in 18 cases: 17 aggressions and 4 cases of hostility (11 male and 7 female), with 9 being assessed as serious.

It also includes the antiepileptic drug perampanel, which was reported in 13 cases of interpersonal violence, including 11 aggresions and 3 angry reactions. Of these, 4 occurred in children and 2 in adolescents; 9 in male and 4 in female.

Eleven cases were reported with methylphenidate: 10 aggressions and one behavioural disturbance (6 were in children, 2 in adolescents, and 3 in adults; 8 of them were male).

With regard to antibacterials for systemic use, 8 cases of aggression related to azithromycin were reported (5 children, 2 infants, and 1 elderly person), of which 7 were male and 1 was a female. With clarithromycin, 6 assaults were found, 4 of which corresponded to the child age group.

The reported cases of interpersonal violence involving antiparkinsonian drugs as suspected medications occurred mainly in male (20 in male and 4 in female), including 14 aggressions, one case of hostility and 3 cases of behavioural disorders.

Regarding the qualitative study of the cases, 96.6% had a compatible time sequence, 75.6% of the cases had well-known ADR, 68% had drug withdrawal and ADR improvement, 0.7% had fatal or irreversible ADR, 96.4% had no or unknown re-exposure. In 32% there was no information about the alternative cause, in 44.4% there was information to rule it out and in 19.2% there was an equally or less plausible explanation.

### 2.5. Case-Non Case

A statistically significant disproportionate reporting was observed with the analgesic tapentadol, the antiepileptics levetiracetam, perampanel, carbamazepine, lamotrigine, topiramate, and valproic acid; the antiparkinsonian drugs carbidopa, levodopa, pramipexole, and rotigotine; the psycholeptics quetiapine, zolpidem, olanzapine, risperidone, haloperidol, clobazam, and hydroxyzine; psychoanaleptics donepezil, fluoxetine, escitalopram, methylphenidate, memantine, duloxetine, atomoxetine, rivastigmine, mianserin, agomelatine and vortioxetine; and other drugs that act on the nervous system bupropion, varenicline and buprenorphine (Table 6 and Supplementary Material).

An association was also detected with drugs not related to the nervous system: systemic hormonal preparations (prednisolone), systemic anti-infectives (azithromycin and clarithromycin), antineoplastic and immunomodulatory agents (interferon alfa-2b and peginterferon alfa-2b) and respiratory system drugs (montelukast) (Table 6 and Supplementary Material).

## 3. Discussion

The present study was conceived with the objective of identifying medications that may be associated with acts of interpersonal violence. This area has been the subject of insufficient exploration and is often poorly understood. The topic is of particular relevance given its sensitive nature and growing social impact, as reflected by recent data indicating that Spain’s homicide rate in 2022 was 0.61 per 100,000 inhabitants [17].

In order to achieve this objective, the study employed a comprehensive and rigorous methodology involving the systematic search and selection of relevant terms from MedDRA^®^ version 24.0 and reports from FEDRA^®^ version 3.0. A high-sensitivity search strategy was implemented, which included both the specific and sensitive Standardised MedDRA Queries (SMQs) for Hostility/Aggression, as well as additional violence-related terms not covered by these SMQs and their corresponding case reports. The inclusion of each term was subjected to rigorous scrutiny to ensure methodological robustness.

The findings of this study indicate that violence-related adverse drug reactions (ADRs) are rarely reported, representing only 0.15% of all ADRs, and are associated with a limited number of medications. This proportion is consistent with observations from previous pharmacovigilance studies conducted in other countries, including France and the United Kingdom [36,44].

Moreover, the majority of reports corresponded to adult and older adult populations, which aligns with prior research by Moore et al. [44] and Rouvé et al. [36]. This finding is not unexpected, as these demographic groups are known to consume a higher volume of medications, which increases their exposure and consequently the likelihood of reporting adverse reactions.

In terms of sex, no differences were observed in the total number of reports of violence. However, males predominated in both the child group (66.3%) and the adolescent group (75.9%). However, the study by Rouve et al. shows a higher percentage of reports in males than in females [36]. Regarding the profile of the notifier, out-of-hospital doctors were the main notifiers.

Thirdly, this study shows the relationship between violence and certain drugs and pharmacological groups, most of which are related to dopamine, serotonin and GABA, whose excess or deficiency could cause psychiatric disorders. The mechanisms of action of these neurotransmitters are fundamental to understanding how chemical imbalances may lead to aggressive or violent behaviours [44,45]. Dopamine, for instance, plays a crucial role in the brain’s reward and motivation pathways; overstimulation of dopaminergic circuits—often induced by psychostimulant drugs—can heighten impulsivity and reduce behavioural control, increasing the risk of aggression. Serotonin, on the other hand, regulates mood, inhibition, and emotional stability; a deficiency in serotonergic activity, whether due to genetic factors or chronic use of certain substances, has been consistently associated with irritability, impulsivity, and violent outbursts. Finally, GABA, the primary inhibitory neurotransmitter in the central nervous system, acts by dampening neural excitability. Substances that suppress GABAergic transmission may lead to heightened arousal and disinhibition, facilitating aggressive responses. Together, these neurochemical pathways illustrate how alterations in neurotransmitter function—whether induced by drug use or underlying psychiatric conditions—can significantly influence violent behaviour.

This study identified a number of medications that were found to be significantly associated with interpersonal violence. These included the anti-Parkinsonian agents carbidopa, levodopa, pramipexole, and rotigotine. The primary mechanism of action of these compounds is through dopaminergic mechanisms, either by enhancing dopamine synthesis or by directly stimulating dopamine receptors. Excessive stimulation of D_2_/D_3_ receptors within mesolimbic pathways has been associated with increased impulsivity, compulsive behaviours, and impaired inhibitory control, which may manifest as aggressive or socially disruptive behaviours. The findings of Rouvé et al. similarly reported associations with the dopamine agonists pramipexole and levodopa, which serves to reinforce the hypothesis that dopaminergic overstimulation can contribute to behavioural dysregulation and aggression [36].

Another pharmacological group that has been identified in reports of interpersonal violence consists of benzodiazepines, with lorazepam, clobazam, and alprazolam being the most frequently reported agents. Of these, clobazam demonstrated a statistically significant association. Although benzodiazepines are primarily known for their anxiolytic and sedative properties, which are mediated by positive allosteric modulation of GABA_A_ receptors, paradoxical reactions have been documented. In susceptible individuals, enhanced GABAergic signalling in specific neural circuits may paradoxically result in a reduction in inhibitory control within cortical–limbic networks, leading to disinhibition, irritability, and aggressive behaviour [46]. These findings are consistent with previous reports describing paradoxical interpersonal violence reactions to benzodiazepines, hypnotics, and sedatives [36,44,47,48].

In addition, the following selective serotonin reuptake inhibitor (SSRI) antidepressants were identified in a number of reports of interpersonal violence: fluoxetine, escitalopram, paroxetine, sertraline, duloxetine and vortioxetine. However, paroxetine and sertraline did not achieve statistical significance. These drugs have been demonstrated to increase synaptic serotonin and modulate a wide range of receptor subtypes (e.g., 5-HT_1A_, 5-HT_2A_, 5-HT_2C_). Although the effects are generally stabilising for mood, they have been observed to precipitate akathisia, agitation or manic episodes in vulnerable individuals. The aforementioned states have been demonstrated to be strongly associated with impulsivity and violent behaviour. Furthermore, it has been hypothesised that dysregulated serotonergic–dopaminergic interactions within limbic circuits may result in heightened emotional reactivity. Consequently, these medications may be associated with various mental disorders, including manic psychosis, and have the potential to induce violence towards others. The association of these antidepressants with violence has been demonstrated in several studies [36,44,49].

The antipsychotic medications reported included olanzapine, quetiapine, risperidone, haloperidol, and aripiprazole, although the latter did not reach statistical significance. Antipsychotics act primarily through antagonism at dopamine D_2_ receptors, often in combination with serotonin receptor modulation. Although these medications are frequently employed in the management of aggression, it is important to note that paradoxical effects may emerge as a consequence. It has been demonstrated that partial dopamine agonism, as evidenced by the administration of aripiprazole, has the potential to induce a state of behavioural instability. This conclusion is consistent with the findings of other studies, including those by Moore et al. [44], which contradicts the results reported by Rouve et al. [36].

The findings of this study also indicated a statistically significant association between interpersonal violence and the antiepileptic drugs sodium valproate, levetiracetam, topiramate, lamotrigine, carbamazepine, and perampanel. It was also reported that pregabalin was identified, but the association was not found to be significant. These drugs modulate diverse mechanisms, including sodium and calcium channel activity, GABAergic signalling, and glutamatergic transmission. Levetiracetam, through its binding to synaptic vesicle glycoprotein 2A (SV2A), has been consistently associated with irritability and aggression. Perampanel, through its antagonism of AMPA receptors, has been strongly linked to hostility and violent outbursts. This is indicative of a disruption of the excitatory–inhibitory balance in cortical–limbic networks. In relation to topiramate, a study by Rouve [36] has demonstrated that the medication can induce hyperactivity and aggression in paediatric patients. However, the study did not identify any instances of violence being associated with topiramate in paediatric populations.

The highest number of cases of interpersonal violence reported were associated with the leukotriene receptor antagonist drug montelukast, and that this drug was suspected of being a cause of ADR. This association was significant, as would have been anticipated, given that several studies had previously demonstrated this evidence and that the adverse reactions listed in its summary of product characteristics (SmPC) include agitation, aggression, anxiety and irritability [44,50,51,52]. While the precise mechanism by which this occurs is not yet fully elucidated, leukotriene receptors have been identified in the brain, and it has been hypothesised that these receptors may influence neuroinflammatory signalling and glutamatergic neurotransmission, thereby impacting mood and impulse regulation.

With regard to antineoplastic and immunomodulatory agents, reports of interpersonal violence were only found for the immunostimulants interferon alfa-2b and peginterferon alfa-2b and the immunosuppressant adalimumab, although statistical significance was not achieved in the case of the latter. Interferons are known to trigger psychiatric adverse reactions largely mediated by pro-inflammatory cytokines (e.g., IL-6, TNF-α) that alter monoamine metabolism and impair emotional regulation. Interferon alfa-2b causes psychiatric adverse reactions such as irritability, agitation, or paranoia, which usually appear after 1 to 3 months and tend to improve within a few days of reducing or withdrawing the drug [36,44].

The only anti-infectives for systemic use reported in notifications of interpersonal violence that showed a significant association were azithromycin and clarithromycin. Aggression is not listed as an adverse reaction in the clarithromycin technical data sheet, although others such as anxiety, psychotic disorders and hallucinations are listed. These effects may result from secondary impacts on mitochondrial function, neuroinflammation, or drug–drug interactions affecting central monoamine systems.

Varenicline and bupropion were associated with a high number of reports of interpersonal violence, also showing a significant causal relationship. In the particular case of varenicline, its link to episodes of aggression and homicidal ideation has been repeatedly documented [36,44], possibly related to its action on α_4_β_2_ nicotinic acetylcholine receptors and modulating dopaminergic transmission in mesolimbic circuits. Bupropion, a norepinephrine and dopamine reuptake inhibitor, enhances catecholaminergic signalling and may precipitate interpersonal violence, particularly in predisposed individuals. Similarly, the psychostimulants methylphenidate and atomoxetine showed significant associations with violence; however, in these drugs, most cases were reported in children and adolescents. These drugs elevate catecholamine tone in the prefrontal cortex and striatum; while this often improves attention and self-control in Attention-Deficit/Hyperactivity Disorder (ADHD), overstimulation can provoke irritability and reactive aggression. These findings are consistent with the results of a study that examined the relationship between these drugs and self-harming behaviour [53].

There are some studies and several case reports describing the onset of mania, psychosis, and symptoms related to interpersonal violence during treatment with efavirenz, a non-nucleoside reverse transcriptase inhibitor, with some patients subsequently requiring treatment with neuroleptics or hospitalisation. Its effects may reflect serotonergic and dopaminergic modulation and psychotomimetic activity resembling NMDA receptor antagonism. Although, only one report has been found in this study and, therefore, no significant association has been found. However, these ADRs, which can be serious, generally disappear when treatment is discontinued [54,55].

Taken together, these findings highlight that aggression and interpersonal violence could arise from complex disruptions in neurotransmission. Hyperdopaminergia promotes impulsivity and compulsive behaviour, serotonergic dysregulation contributes tomanic states, GABAergic disinhibition may result in paradoxical aggression, glutamatergic imbalance destabilises cortical–limbic excitatory control, and neuroimmune signalling alters monoaminergic regulation. The convergence of these mechanisms underscores the multifactorial neurobiology of pharmacologically induced aggression.

Although these adverse reactions are described for virtually all of these drugs in their Summary of Product Characteristics (SmPC), it is necessary to emphasise them in order to improve their prescription with the aim of preventing them.

Our study is subject to certain limitations inherent in studies based on spontaneous reports of adverse drug reactions. The primary cause of this discrepancy is under-reporting in the spontaneous reporting pharmacovigilance system, particularly in cases of interpersonal violence. It is challenging for patients, especially those with chronic psychiatric illness or a history of violence, to report the suspicion of an association between a drug and a violent act, resulting in an underestimation of the problem’s severity. Furthermore, the reporting rate may be subject to variation depending on the type and severity of the reaction, the drugs involved, and the categorisation of the drug as a new or existing pharmaceutical compound. Conversely, FEDRA^®^ reports utilise a series of standardised medical terms to describe each report. The section where the finding can be explained narratively is often not filled in, resulting in variations in the quality and detail of the reports. It is also necessary to mention the lack of sufficient data in most reports on family and/or individual history of violence, alcohol and drug use, and dosage of the medication used.

It is important to note that the inherent nature of a retrospective design carries with it the potential for bias, including selection bias, incomplete or missing data, and the lack of control over confounding variables. Furthermore, factors associated with the patients’ underlying diseases or variations in clinical management may have influenced the observed outcomes. These limitations may have implications for the reliability and generalisability of the findings. Consequently, while they facilitate the identification of potential associations, a more comprehensive evaluation is imperative [56]. Furthermore, it is recommended that subsequent research endeavours encompass the utilisation of more contemporary data, thereby facilitating the evaluation of potential temporal fluctuations in the reporting of adverse drug reactions that are concomitant with interpersonal violence. On the other hand, studies should be conducted in children and adolescents, as these are particularly sensitive population groups who are prescribed medications clearly related to interpersonal violent behaviour.

Despite all the limitations, like quantitative signal detection methods, they are increasingly used both for signal generation in pharmacovigilance and for drug safety research, as they greatly facilitate the identification of new safety issues or possible harmful effects of a product [57,58,59,60,61,62,63]. Thus, these methods contribute to the protection of public health by detecting rare adverse reactions to medicines, while covering all medicines available on the market. Moreover, these results underscore the importance of careful monitoring, particularly in vulnerable populations and in individuals with a history of psychiatric disorders.

## 4. Materials and Methods

A descriptive, longitudinal and retrospective study and case/non-case study of spontaneous reports of adverse drug reactions (ADRs) corresponding to interpersonal violence, included in the database of the Spanish Human Pharmacovigilance System (FEDRA^®^ 3.0). This study included all reports from 1984 to 31 March 2021. Physical, psychological, verbal and thought acts are defined as interpersonal violence.

### 4.1. Selection of Cases

In the initial phase of the study, the preferred terms (PT) from the Medical Dictionary for Regulatory Affairs (MedDRA 24.0^®^) that most accurately described interpersonal violence-related reactions were identified. Subsequently, spontaneous reports containing these terms were retrieved and selected from the FEDRA^®^ 3.0 database.

For this purpose, the Standardised MedDRA Queries (SMQ) Narrow Hostility/Aggression were used, which includes the PTs physical abuse, sexual abuse, aggression, physical assault, conduct disorder, belligerence, antisocial behaviour, abusive behaviour towards a child, homicide, hostility, homicidal ideation, incest, psychopathic personality, anger reaction, violence-related symptom, antisocial personality disorder, intermittent explosive disorder, borderline personality disorder. In addition, the SMQ Hostility/Aggression sensitive PTs abuse of an elderly person, spousal abuse, psychological abuse, verbal abusive behaviour, delinquency, agitated depression, shouting, gunshot wound, stab wound, bite, human bite, paraphilia, paranoia, paedophilia, fighting at school, pyromania, robbery, sadism, activation syndrome, bipolar disorder I, bipolar disorder II, personality disorder, impulse control disorder, delusional disorder, jealous type, delusional disorder, persecution type, delusional disorder, jealous type, bipolar disorder, oppositional defiant disorder, and paranoid personality disorder were included.

All reports containing one or more MedDRA terms (PT) were searched for, identified and evaluated. Thereafter, ADRs were revised. When the reporting form was not sufficiently informative or when the characteristics of ADRs could not be completely defined, the report was not included in the analysis.

### 4.2. Statistical Analysis

#### 4.2.1. Descriptive Study

Descriptive statistical analyses were conducted for each variable to characterise ADRs of interpersonal violence, using SPSS Statistics for Windows, Version 19. (IBM Corp., Armonk, NY, USA).

Among the variables included in the reports, the following were selected for analysis:

- General data on reports: date and seriousness of the report.
- Primary sources: physicians, pharmacists, nurses, other health professionals and consumers, as well as from in-hospital or out-of-hospital.
- Patient data: sex, age, age group. The age groups are infant (0–1.9 years old), child (2–11 years), adolescent (12–17 years), adult (8–65 years) and elderly (over 65 years).
- ADRs: reactions of interpersonal directed violence (several ADRs can be reported in one report), which were analysed according to PT, High Level Term (HLT) and System Organ Class (SOC), as well as severity and outcome of ADRs.
- Drugs: drug name, suspected or concomitant drug. Suspected drugs were analysed according to the Anatomical Chemical Therapeutic Classification System and a qualitative analysis was carried out including time sequence, prior knowledge, re-exposure and alternative cause.

#### 4.2.2. Case-Non Case Method

This method is based on measures of disproportionality between a drug and the ADR of interest in the Spanish Human Pharmacovigilance Database. Cases are the notifications corresponding to the ADR of interest (interpersonal violence) and non-cases are all other notifications in FEDRA^®^ 3.0 database.

The strength of the association was analysed for drugs that had three or more reports of interpersonal violence.

Three statistics were used to quantify the strength of the association between exposure to the drug and the occurrence of the adverse reaction: Reporting Odds Ratio (ROR) (95% CI of ROR > 1), Ratio of Proportions of Reports (PRR > 2), Chi square (with Yates correction) (X^2^ > 4).

## 5. Conclusions

The results of the study indicate that, although interpersonal violence is a rare occurrence, it has the potential to manifest as a clinically significant adverse reaction in a small subset of medications. The majority of reported suspected ADRs of interpersonal violence were classified as serious, with the majority of these cases affecting adults and the elderly, and no discernible sex differences except in child and adolescent age groups. The principal drugs associated with interpersonal violence were montelukast, levetiracetam, bupropion, donepezil, perampanel, quetiapine, fluoxetine, and lorazepam. A statistically significant association/disproportion in the notification has been identified in the reporting of interpersonal violence and different drugs according to the literature, notably atomoxetine, perampanel, memantine, donepezil, montelukast and methylphenidate. Despite the absence of unanticipated outcomes, this study has the potential to reinforce the scientific evidence supporting the association between interpersonal violence and drug use. The extant literature predominantly concentrates on interpersonal violence in the context of pharmaceutical documentation, labelling, academic literature and associated domains. Nevertheless, epidemiological and clinical data remain scarce. It has also been demonstrated that this can assist in increasing awareness among healthcare providers, who, it is recommended, should acknowledge this link in order to prevent or mitigate its effects. Furthermore, research should place particular emphasis on children and adolescents, as they constitute a vulnerable group that is frequently prescribed drugs with a demonstrable association with violent behaviour. It is evident that further research is required, incorporating the utilisation of prescribed medications within a comprehensive framework that encompasses the multifaceted factors influencing interpersonal violence. This framework must encompass social, biological, economic, political, cultural, and educational dimensions.

## Figures and Tables

**Table 1:** Distribution of interpersonal violence reports in FEDRA^®^ according to Preferred Term of the MedDRA classification.

Preferred Terms of the MedDRA Classification	N *	Percentage
Aggression	480	86.6
Behavioural disturbance	35	6.3
Anger reaction	19	3.4
Hostility	10	1.8
Symptom related to violence	4	0.7
Homicidal ideation	3	0.5
Antisocial behaviour	2	0.4
Intermittent explosive disorder	1	0.2
Total	554	100.0

* Number of reports of interpersonal violence reported in FEDRA^®^.

**Table 2:** Outcome of interpersonal violence adverse drugs reactions.

Outcome	N *	Percentage
Not reported	105	19.0
Recovering	39	7.0
Fatal	3	0.5
Not recovered	47	8.5
Recovered	353	63.7
Recovered with sequelae	7	1.3
Total	554	100.0

* Number of reports of interpersonal violence reported in FEDRA^®^.

**Table 3:** Distribution of interpersonal violence reports in FEDRA^®^ according to Anatomical Therapeutic Classification (ATC).

ATC First Level/Therapeutic Group		N *	Percentage
A	Alimentary tract and metabolism	25	3.2
B	Blood and blood-forming organs	5	0.7
C	Cardiovascular system	14	2.1
D	Dermatologicals	2	0.2
G	Genito urinary system and sex hormones	15	2.4
H	Systemic hormonal preparations, excluding sex hormones and insulins	16	2.2
J	Antiinfective for systemic use	67	10
L	Antineoplastic and immunomodulating agents	26	3.9
M	Musculo-skeletal system	13	1.6
N	Nervous system	419	62.3
P	Antiparasitic products, insecticides and repellents	8	1.2
R	Respiratory system	57	8.6
S	Sensory organs	3	0.4
V	Various/several	1	0.1
Total		671	100

* Number of reports of interpersonal violence reported in FEDRA^®^.

**Table 4:** Distribution of interpersonal violence reports of Nervous System drugs.

Nervous System		N *	Percentage
N01	Anaesthetics	5	0.7
N02	Analgesics	27	4.2
N03	Antiepileptics	84	12.8
N04	Antiparkinsonians	32	4.7
N05	Psycholeptics	105	15.3
N06	Psychoanaleptics	126	18.8
N07	Other nervous system drugs	40	5.8
Total		419	62.3

* Number of reports of interpersonal violence reported in FEDRA^®^.

**Table 5:** Prescribe/Individual drugs most often involved in case reports of interpersonal violence in the Spanish PharmacoVigilance Database (n ≥ 8).

DRUG	N *	%
Montelukast	35	5.2
Levetiracetam	23	3.4
Bupropion	18	2.7
Donepezil	14	2.1
Perampanel	13	1.9
Quetiapine	13	1.9
Fluoxetine	13	1.9
Lorazepam	12	1.8
Zolpidem	11	1.6
Escitalopram	11	1.6
Methylphenidate	11	1.6
Memantine	10	1.5
Varenicline	10	1.5
Olanzapine	9	1.3
Azithromycin	8	1.2
Duloxetine	8	1.2

* Number of reports of interpersonal violence reported in FEDRA^®^.

**Table 6:** Drugs most statistically significant implicated in FEDRA^®^ reports of interpersonal violence and analysis of disproportionate occurrence calculated using the case/non-case method.

Drug	N ^1^	ROR (IC 95%)	PRR (IC 95%)	χ^2^ **
**Nervous System**				
*Analgesics*				
Tapentadol	5	4.83 (2.00–11.69) *	4.80 (2.00–11.55) *	11.26 *
*Antiepileptics*				
Levetiracetam	23	12.65 (8.30–19.28) *	12.44 (8.22–18.82) *	216.31 *
Perampanel	13	59.14 (33.34–104.93) *	54.48 (32.12–92.40) *	568.01 *
Lamotrigine	5	3.26 (1.35–7.88) *	3.25 (1.35–7.82) *	5.59 *
Topiramate	5	2.67 (1.10–6.44) *	2.66 (1.10–6.41) *	3.58 *
*Antiparkinsonians*				
Levodopa	7	3.42 (1.62–7.23) *	3.41 (1.62–7.18) *	9.43 *
Pramipexole	7	11.66 (5.50–24.74) *	11.48 (5.48–24.04) *	55.07 *
Carbidopa	6	3.49 (1.56–7.82) *	3.48 (1.56–7.76) *	8.08 *
Rotigotine	4	9.38 (3.48–25.26) *	9.26 (3.49–24.61) *	21.29 *
*Psycholeptics*				
Quetiapine	13	6.18 (3.56–10.75) *	6.14 (3.55–10.61) *	49.06 *
Zolpidem	11	4.82 (2.65–8.77) *	4.79 (2.64–8.68) *	28.41 *
Olanzapine	9	5.18 (2.67–10.04) *	5.15 (2.67–9.93) *	25.38 *
Risperidone	7	2.85 (1.35–6.01) *	2.84 (1.35–5.97) *	6.45 *
Haloperidol	5	3.59 (1.48–8.67) *	3.57 (1.48–8.60) *	6.73 *
Clobazam	4	9.55 (3.55–25.72) *	9.43 (3.55–25.04) *	21.77 *
*Psychoanaleptics*				
Donepezil	14	19.47 (11.36–33.34) *	18.95 (11.23–31.99) *	208.43 *
Fluoxetine	13	3.79 (2.18–6.58) *	3.78 (2.18–6.54) *	23.03 *
Escitalopram	11	3.42 (1.88–6.23) *	3.41 (1.88–6.19) *	15.92 *
Methylphedate	11	13.97 (7.64–25.54) *	13.71 (7.59–24.76) *	112.28 *
Memantine	10	22.09 (11.70–41.71) *	21.42 (11.57–39.65) *	165.89 *
Duloxetine	8	3.16 (1.57–6.37) *	3.15 (1.57–6.33) *	9.47 *
Atomoxetine	7	31.48 (14.68–67.51) *	30.11 (14.53–62.43) *	159.31 *
Rivastigmine	5	5.28 (2.18–12.79) *	5.25 (2.18–12.62) *	12.94 *
*Other nervous system drugs*				
Bupropion	18	11.19 (6.97–17.97) *	11.03 (6.92–17.58) *	146.34 *
Varenicline	10	8.79 (4.69–16.51) *	8.69 (4.67–16.19) *	58.58 *
Buprenorphine	5	3.49 (1.44–8.44) *	3.48 (1.44–8.37) *	6.40 *
**Non-nervous system**				
*Systemic hormonal preparations, excluding sex hormones and insulins*				
Prednisolone	4	6.28 (2.34–16.88) *	6.23 (2.34–16.59) *	12.48 *
*Systemic anti-infectives*				
Azithromycin	8	2.72 (1.35–5.47) *	2.71 (1.35–5.44)	6.83 *
Clarithromycin	6	2.30 (1.03–5.16) *	2.30 (1.03–5.30) *	3.14
*Antineoplastic and immunomodulating agents*				
Interferon Alfa-2B	4	7.70 (2.86–20.71) *	7.62 (2.87–20.27) *	16.50 *
*Respiratory system*				
Montelukast	35	17.85 (12.62–25.25) *	17.43 (12.42–24.46) *	478.66 *

* *p* < 0.05 ROR: Reporting odds ratio. PRR: Reporting Proportion Ratio. ** χ^2^: Chi square (with Yates correction). ^1^ Number of reports of interpersonal violence reported in FEDRA^®^.

## Data Availability

The original contributions presented in this study are included in the article/Supplementary Materials. Further inquiries can be directed to the corresponding author.

## Supplementary Materials

The following supporting information can be downloaded at: https://www.mdpi.com/article/10.3390/ph18121845/s1, File S1: Complete list of interpersonal violence reports for each of the 251 drugs.
